# Adolescents' social anxiety dynamics in a latent transition analysis and its psychosocial effects

**DOI:** 10.1016/j.ijchp.2022.100311

**Published:** 2022-05-27

**Authors:** Antonio Camacho, Rosario Ortega-Ruiz, Eva M. Romera

**Affiliations:** Universidad de Córdoba, Spain

**Keywords:** Latent profile analysis, Spanish adolescents, Peer adjustment, Peer victimization, Subjective well-being

## Abstract

**Background/Objective:**

The present study aims to explore the dynamics of social anxiety profiles in adolescents over time and the psychosocial effects these dynamics have.

**Method:**

A representative sample of Andalusian (southern Spain) adolescents in Secondary Education was drawn. The study used single-stage stratified cluster sampling. A total of 2,140 students aged 11-16 years (47% girls; *M_age_*T1 = 13.68, *SD* = 1.27) were involved at two time points with a six-month interval.

**Results:**

The results provided a four-profile structure: low social anxiety, moderate cognitive disturbance, high with difficulties in new situations, and high social anxiety. The latent transition analysis showed a stability in the social anxiety profiles of between 58%-61%. Those adolescents who remained in or transitioned to profiles with higher social anxiety scored worse on peer adjustment, peer victimization and subjective well-being.

**Conclusions:**

The study may contribute the improvement of the psychological treatments in social anxiety and reduce adverse effects on peer relationships and well-being by distinguishing the profiles and their dynamics.

Social anxiety refers to fear or stress about social situations or to be negatively evaluated by others. According to three-dimensional theory (Lang, 1968), social anxiety emerges through a three-dimensional response system (cognitive, emotional, and behavioral). The first dimension relates to the fear of being negatively evaluated by others, and the other two dimensions reflect social avoidance and distress in (a) new situations and (b) in general (La Greca & Lopez, 1998). A recent study questions this traditional classification in favor of comprehensive patterns of social anxiety in which cognitive, behavioral, and emotional responses all play a role to different degrees (Yu et al., 2020).

The research into social anxiety has developed mostly a person-centered approach, in which individuals are categorized into subgroups. In studies with adolescents, most classifications were made along a unidimensional scale of social anxiety (Chiu et al., 2021). Only one study (Yu et al., 2020) has analyzed the heterogeneity of social anxiety by using the cognitive, emotional, and behavioral responses in a latent profile analysis (LPA). The study applied the commonly used Social Anxiety Scale for Adolescents (La Greca & Lopez, 1998), and found a four-profile model (low social anxiety, moderate cognitive disturbance, moderate difficulties in new situations, and high social anxiety. Compared to the low group, more girls and later adolescents were found in the groups with moderate to high social anxiety.

Higher levels of social anxiety have been associated with poorer peer functioning (see meta-analysis by Chiu et al., 2021), which in turn may increase the severity of social anxiety. Maladaptive effects of social anxiety have been consistently found for peer adjustment (Early et al., 2017; Gómez-Ortiz et al., 2020; Ooi et al., 2017), peer victimization (Chiu et al., 2021; Romera et al., 2022) and subjective well-being (Fernandez et al., 2018). Most studies that have looked at the influence of social anxiety on psychological and peer outcomes have used a variable-centered approach (e.g., correlation and regression analyses, and structural equation modeling). This approach assumes that the effects of social anxiety on psychosocial factors operate homogeneously across adolescents, whereas studying these psychosocial effects needs to incorporate the heterogeneity of social anxiety, as is done with a person-centered approach.

The current study aims to replicate the Yu et al.'s (2020) approach in a Spanish sample. Social anxiety is influenced by the cultural context (Rapp et al., 2021) and higher levels of social anxiety, based on the same instrument, have been found among Chinese adolescents in comparison to their Spanish peers (Zhou et al., 2008). Adding insights into the heterogeneity of social anxiety in a different cultural setting is therefore valuable in itself. The present study, however, goes further by also using this heterogeneous approach to social anxiety to investigate the links with previously identified maladaptive effects on peer adjustment, peer victimization and subjective well-being.

All studies mentioned above used cross sectional data, but to understand the dynamics of social anxiety profiles over time and to explore how this is associated with adolescents' psychosocial adjustment requires a longitudinal design. The present article contributes to the literature to apply LTA by studying changes in adolescents' profiles that are based solely on social anxiety. Applying this approach to social anxiety can guide the design of preventive interventions. Current prevention programs predominantly focus on cognitive behavior or behavior, and do not consider the dynamic interplay between profiles (Hoffman y DiBartolo, 2014). New interventions with diversified strategies that incorporate not only the differences between social anxiety profiles, but also how adolescents transit between these profiles over time are therefore needed.

This study aims to identify different social anxiety patterns by taking individual variation in cognitive, emotional, and behavioral responses into account at two time points. The study was limited to early and middle adolescence because it is a key developmental stage on the effects of social anxiety on the psychosocial adjustment of students (Peleg, 2012), a key stage period in which the peer group acquires a great relevance, through support and learning, but also as a source of social pressure and anxiety. In the present study, gender, age, and social demonstration-avoidance goals were included in the analyses as covariates (both time points), because girls, middle adolescents and those with higher levels of social demonstration-avoidance goals are expected to be more often found in the profiles with more severe social anxiety (Gómez-Ortiz et al., 2020; Yu et al., 2020). As evidenced previously on the effects of social anxiety on psychosocial adjustment (Fernandez et al., 2018; Ooi et al., 2017; Romera et al., 2022), peer victimization, peer adjustment and subjective well-being are included in the second time point to explore whether there are differences in those adolescents who move towards or stay in profiles with lower social anxiety. It also expected that those adolescents who move towards higher social anxiety profiles show poorer psychosocial adjustment (higher involvement in peer victimization and lower peer adjustment and subjective well-being) compared to those who remain in or move to the lower social anxiety profiles.

## Method

### Participants

To obtain a representative sample of Andalusian (South of Spain) middle school adolescents, the study used single-stage stratified cluster sampling with two strata: Western and Eastern Andalusia, and the probability of selecting clusters (all public and private middle schools) proportional to the total number of schools within each stratum. In schools where each grade had more than one class, the class that participated in the study was randomly allocated. A total of 2,183 adolescents were called to participate in the study, of whom 2,140 (47% girls) agreed to participate and were consented by their parents or guardians. Participants were between 11 and 16 years old from grades 7 (24%), 8 (26%), 9 (25%) and 10 (25%) from public (62%) and private middle schools (38%). In roughly equal parts, participants were from towns with less than 10,000 inhabitants (34%), between 10,001 and 100,000 (34%), and more than 100,000 inhabitants (31%). The questionnaires were administrated twice with a six-month interval, first in October 2017 (Time 1 – T1 hereafter: *n* = 1,696, 79% participation rate; *M*_age_ = 13.68, *SD* = 1.27) and then in May 2018 (T2: *n* = 1,929, 90% participation rate, *M*_age_ = 14.10, *SD* = 1.30).

### Measures

The Spanish version (Olivares et al., 2005) of the Social Anxiety Scale for Adolescents (La Greca & Lopez, 1998) was used at T1 and T2. The scale includes 18 items structured according to the three dimensions of social anxiety (“I worry about what others say about me”) from 1 (*Not at all*) to 5 (*All the time*). Reliability in the original study was good (α = .80 - .94; Olivares et al., 2005). In the present study, the internal consistency was ω_T1_ = .80 - .89 andω_T2_ = .77 - .89. A confirmatory factor analysis showed the current data adjusted properly to this three-factor structure: χ²/*df* = 6.91, *p* < .001; CFI = .920, TLI = .914, RMSEA = .056, 90% CI [.054, .058], SRMR = .068.

The peer adjustment subscale of the Adolescent Multidimensional Social Competence Questionnaire (Gómez-Ortiz et al., 2017) was used at T2. The subscale includes 8 items (“My classmates help me when I need it”) from 1 (*Totally false*) to 7 (*Totally true*). Reliability in the original study was excellent (ω = .91; Gómez-Ortiz et al., 2017). The internal consistency in the current study was good ($\omega_{T2}=.92$). A confirmatory factor analysis of the current data showed they adjusted properly to the unidimensional structure: χ²/*df* = 9.12, *p* < .001; CFI = .971, TLI = .967, RMSEA = .066, 90% CI [.062, .070], SRMR = .034.

The victimization subscale of the European Bullying Intervention Project Questionnaire (Ortega-Ruiz et al., 2016) was used at T2. The scale includes 7 items (“I was excluded or ignored by others”). Adolescents scored items from 0 to 4 (0 = *no*; 1 = *once or twice*; 2 = *once or twice a month*; 3 = *once a week*; and 4 = *more than once a week*) based on the frequency with which they experienced victimization behaviors in the previous three months. Reliability in the original study was good (α = .80; Ortega-Ruiz et al., 2016). The internal consistency of the subscale in the current study was good ($\omega_{T2}=.86$). A confirmatory factor analysis of the current data showed they adjusted properly to the unidimensional structure: χ²/*df* = 6.11, *p* < .001; CFI = .965, TLI = .959, RMSEA = .052, 90% CI [.048, .057], SRMR = .047.

The Spanish version (Casas et al., 2012) of the Personal Well-being Index (Cummins et al., 2003) was used at T2. The scale includes 7 items asking adolescents about the level of satisfaction with different domains of their lives (health, life achievements, security for the future and interpersonal relationships) from 1 (*Completely dissatisfied*) to 10 (*Completely satisfied*). Reliability in the original study was good (α = .80; Casas et al., 2012). The internal consistency in the current study was good ($\omega_{T2}=.89$). A confirmatory factor analysis of the current data showed they adjusted properly to the unidimensional structure: χ²/*df* = 8.98, *p* < .001; CFI = .964, TLI = .961, RMSEA = .065, 90% CI [.061, .070], SRMR = .033.

The Spanish version of the social demonstration-avoidance subscale (Herrera-López et al., 2016) of the Social Achievement Goal Scale (Ryan & Shim, 2006) was used at T1 and T2. The subscale includes 4 items (“I try not to goof up when I am out with people”) from 1 (*Totally false*) to 5 (*Totally true*). Reliability in the original study was good (α = .81; Herrera-López et al., 2016). The internal consistency in the current study was good ($\omega_{T2}=.72\;and\;\omega_{T2}=.92$). A confirmatory factor analysis showed the current data adjusted properly to the unidimensional structure: χ²/*df* = 9.09, *p* < .001; CFI = .965, TLI = .956, RMSEA = .066, 90% CI [.057, .074], SRMR = .034.

### Procedure

The current study was approved by the Ethics Committee of the authors’ institution. After receiving approval from the schools' management staff, permission from the regional government and parental informed consent were obtained. The participants completed the paper and pencil questionnaires during their lessons anonymously and confidentially. Participation was voluntary and students could leave at any time. On average, it took 30 minutes to answer the questions.

### Data analyses

The analysis followed a five-step procedure (Nylund, 2007). In Step 1, one to five latent profiles based on the responses to the 18 SAS-A items were examined independently through latent profile analysis (LPA), and then compared. Better model fit is indicated by lower values of Akaike's Information Criterion (AIC), Bayesian Information Criterion (BIC) and Sample-size adjusted Bayesian Information Criterion (SABIC). The Vuong-Lo-Mendell-Rubin (VLMR) was run to compare *k* to *k*-1 profile solutions, in which a significant *p* value indicates a better fit of the *k* profile solution. Entropy was tested to assess how accurately adolescents were clustered in the different groups. After determining the optimal number of profiles, gender, age, and social demonstration-avoidance goals were examined as covariates of the social anxiety profiles. In Step 2, measurement invariance of the LTA across T1 and T2 was analyzed to explore the consistency of the different social anxiety profiles. First, a base LTA was freely estimated without constraints. Then, parameters were restricted to be equal across time, and the fit indices of both models were assessed in a log likelihood ratio test (LRT). In Step 3, the profile transition matrix from T1 to T2 was obtained through LTA. In Step 4, covariates were added in the LTA separately in a 3-stage approach by testing the conditional transition probabilities through the delta method at low and high values (±1 *SD*) for the social demonstration-avoidance goals variables, while gender (1 = boys; 2 = girls) and age (1 = early adolescents: 11 - 13 years; 2 = middle adolescents: 14 - 16 years) were included as dichotomous variables. In Step 5, the “Model Constraint” procedure with paired-sample *t* tests was used to analyze differences in peer adjustment, peer victimization and subjective well-being at T2 about the profile transitions. Analyses were run in M*plus* 8.7 (Muthén & Muthén, 1998) and used a robust maximum likelihood (MLR) estimator.

Little's MCAR test showed that the data were not completely missing at random (*p* < .001) (MCAR) but were missing at random (MAR) as indicated by the low normalized chi-squared result (χ^2^/*df* = 1.30). Accordingly, all individuals were included in the analyses with the data provided via full information maximum likelihood estimation (FIML).

## Results

### Measurement of alternative models

The fit indices from the LPA model solutions with one to five profiles are shown in Table 1 at T1 and T2. At both time points, the information criteria (AIC, BIC and SABIC) improved as the number of profiles increased. The entropy indicated a high classification precision for each profile (> .875 at T1; > .888 at T2). Based on the *p* values from the VLMR test, the four-profile solution was the most parsimonious solution, as the five-profile solution offered no significant improvement of the fit indices. The class membership probabilities for each profile in the four-profile solution provided good discrimination and reliability (from 92% to 95% at both times).

**Table 1:** Fit Indices for latent profile models with 1 to 6 profiles at two time points and profile prevalence.

Profiles	m	LogL	AIC	BIC	SABIC	VLMR	Entropy	1	2	3	4	5	6
Time 1													
1 profile	72	-47,278.855	94,701.711	95,101.924	94,873.179	-	-	-	-	-	-	-	-
2 profiles	145	-43,396.427	87,082.853	87,888.838	87,428.172	< .001	.907	.45	.55	-	-	-	-
3 profiles	218	-42,243.613	84,923.226	86,134.983	85,442.394	< .001	.887	.44	.20	.36	-	-	-
**4 profiles**	**291**	**-41,558.315**	**83,698.631**	**85,316.159**	**84,391.649**	**.0432**	**.875**	**.25**	**.31**	**.26**	**.18**	-	-
5 profiles	364	-41,019.620	82,767.241	84,790.541	83,634.109	.1748	.878	.29	.21	.14	.14	.22	-
Time 2													
1 profile	72	-41,633.492	83,410.984	83,802.037	83,573.303	-	-	-	-	-	-	-	-
2 profiles	145	-38,239.460	76,768.919	77,556.458	77,095.811	< .001	.888	.43	.57	-	-	-	-
3 profiles	218	-37,021.389	74,478.778	75,662.801	74,970.244	< .001	.890	.39	.18	.43	-	-	-
**4 profiles**	**291**	**-36,302.808**	**73,187.617**	**74,768.125**	**73,843.656**	**.0552**	**.890**	**.28**	**.27**	**.31**	**.15**	-	-
5 profiles	364	-35,754.943	72,237.886	74,214.879	73,058.498	.7667	.894	.14	.15	.24	.25	.23	-

**Fig. 1:**
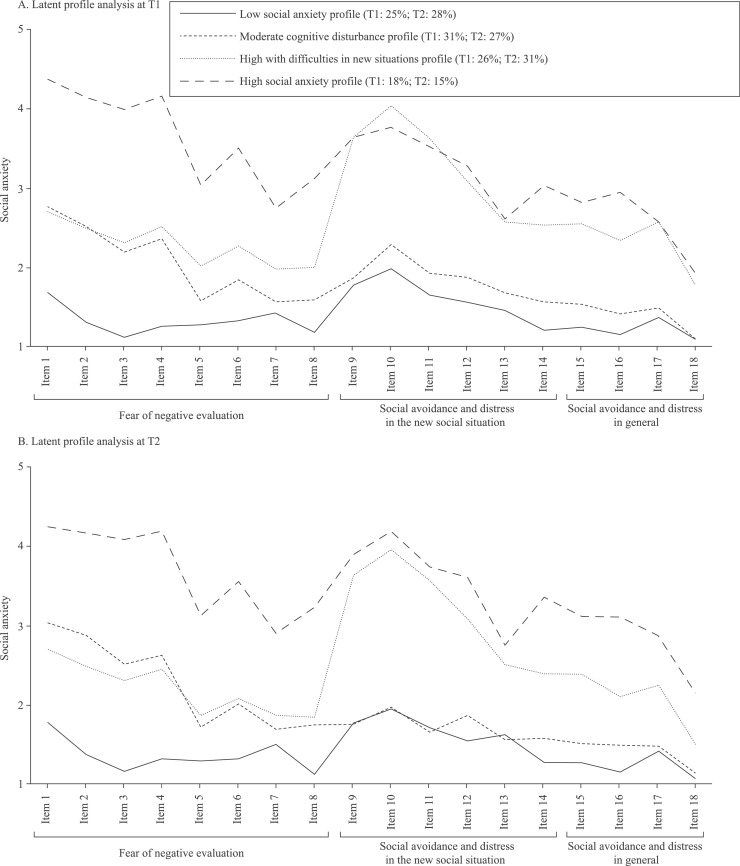
Latent Profile Analyses for T1 and T2.

The interpretation of the profiles was based on the three dimensions of the social anxiety scale, as can be seen in Fig. 1 (Fig. 1A for T1; Fig. 1B for T2). The adolescents in profile 1 were labelled as “low social anxiety” (LSA) based on the general pattern of low scores. Profile 2 was characterized by moderate values on fear of negative evaluation and low levels of social avoidance and distress in new situations and in general, leading to a label of “moderate cognitive disturbance” (MC). The high values in avoidance and distress in new situations of profile 3 led to label this profile as “high with difficulties in new situations” (HNS). Finally, profile 4 was labeled as “high social anxiety” (HSA) based on the general pattern of high scores across the scale compared to other profiles.

LPA with covariates were run for each time point (see Table 2), using the low social anxiety profile as the reference case. Being female increased the likelihood of being clustered in the moderate and high social anxiety profiles. Older adolescents were more likely to be found in the moderate and high profiles at T1 and in the HNS profile at T2. Having higher social demonstration-avoidance goals was a risk factor for being in the higher social anxiety profiles.

**Table 2:** Odds ratio results of the conditional latent profile analyses examining individual effects at T1 and T2.

	LSA^1^		
	MC	HNS	HSA
Time 1			
Gender (1 = boy, 2 = girl)	.74*	.54***	.40***
Age	.82**	.76***	.82*
Social demonstration-avoidance goals	.44***	.34***	.13***
Time 2			
Gender (1 = boy, 2 = girl)	.58***	.63***	.44***
Age	.97	.77***	.87
Social demonstration-avoidance goals	.92	.40***	.18***

Note. * p < .05, ** *p* < .01, *** *p* < .001.

1 Reference profile. LSA = Low social anxiety; MC = Moderate cognitive disturbance; HNS = High with difficulties in new situations; HSA = High social anxiety.

### Measurement invariance

This step revealed no significant differences between the model fit indices between the constrained model (log-likelihood = -77.547.264; AIC = 156,276.528; BIC = 159.624.988; SABIC = 157,747.312) and the unconstrained model (log-likelihood = -77,726.730; AIC = 156,059.459; BIC = 157,776.183; SABIC = 156,813.516). The LRT (*x*^2^_(_*_df_*_= 288)_ = .51, *p* > .05), suggesting the same number and type of profiles are reliable across the two time points. The next steps were therefore performed with the constrained model.

### Latent transition model without covariates

**Fig. 2:**
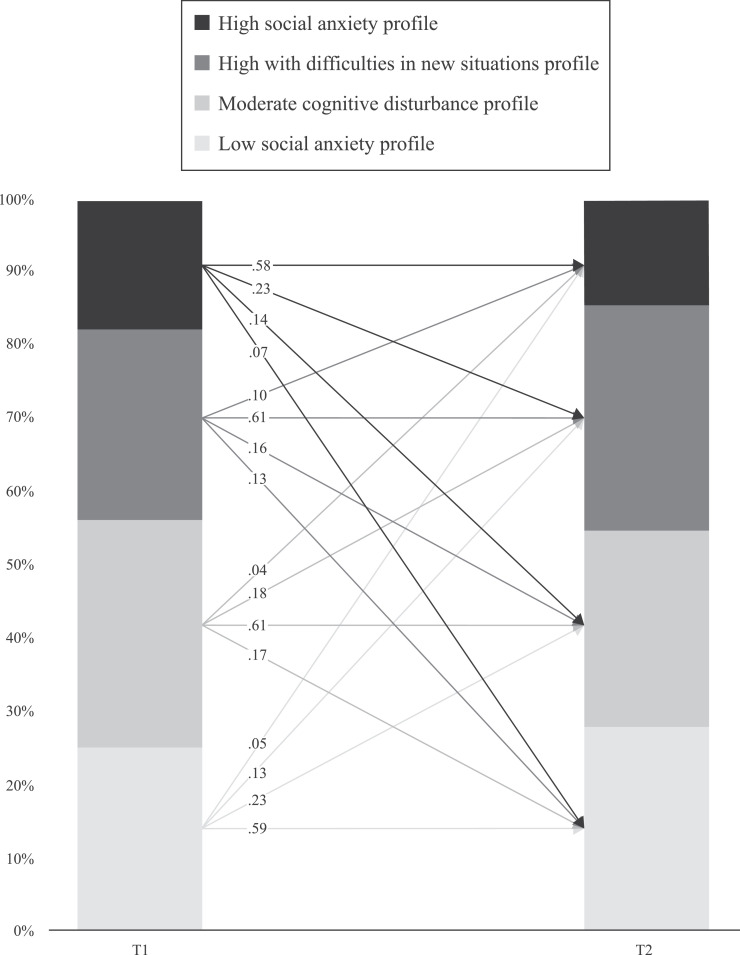
Transition Probabilities from T1 to T2.

Fig. 2 presents the transition probabilities for each profile from T1 to T2. The results indicated a moderate level of stability in the profiles (58% - 61%). For adolescents in the LSA profile at T1, the likelihood of changing to another profile decreases for profiles with greater social anxiety. Similarly, the probability of transitioning from the HSA profile at T1 to other profiles at T2 decreases for profiles with lower social anxiety. The probability to transition from the MC profile at T1 to the LSA or HSN profiles at T2 is higher than the probability to transition to the HSA profile. Finally, the probability of transitioning from the HSN profile at T1 to the MC profile at T2 is higher than transitioning to the LSA profile, which in turn is higher than the probability to transition to the HSA profile.

### Latent transition model with covariates

Checking for gender differences in the transition probabilities showed that boys were more likely than girls to move from: (a) the low to the high social anxiety profile (9% boys vs. 1% girls; *t* = -2.14, *p* < .05); (b) the high to the low social anxiety profile (14% boys vs. 3% girls; *t* = -3.06, *p* < .01). Greater stability was found for girls, by remaining within the MC (55% boys vs. 69% girls; *t* = 2.27, *p* < .05) and the HSA profile (49% boys vs. 63% girls; *t* = 1.98, *p* < .05). There were neither differences between early and middle adolescents in any of the transition probabilities, nor were there between high and low levels of social demonstration-avoidance goals.

### Outcomes

After controlling for the effects of covariates in the LTA, the psychosocial implications at T2 on peer adjustment, peer victimization and subjective well-being of the transitions between profiles were examined. This was done for each T1-profile separately, each time using the transition to the LSA profile as a reference. The results are reported in Table 3.

**Table 3:** Psychosocial outcomes of the different profile transitions compared.

	*M* (*SD*)				*t*-tests (Cohen’ *d*)			
Transition pattern	PA	PV	SWB			PA	PV	SWB
**LSA (no transition)**	**5.70 (1.32)**	**.41 (.29)**	**8.74 (.174)**			-	-	-
LSA → MC	5.66 (.96)	.56 (.33)	8.62 (1.33)			ns	ns	ns
LSA → HNS	5.25 (1.35)	.32 (.11)	7.58 (5.27)			ns	ns	-2.87** (.46)
LSA → HSA	5.45 (2.00)	.84 (.77)	8.82 (1.81)			ns	ns	ns
**MC** → **LSA**	**5.62 (1.15)**	**.35 (.33)**	**8.83 (1.18)**			-	-	-
MC (no transition)	5.86 (.60)	.34 (.14)	8.93 (.58)			ns	ns	ns
MC → HNS	5.24 (1.01)	.82 (.57)	8.50 (.89)			ns	ns	ns
MC → HSA	4.95 (1.36)	.17 (.22)	8.03 (1.32)			ns	ns	ns
**HNS** → **LSA**	**5.82 (1.37)**	**.13 (.03)**	**8.14 (3.00)**			-	-	-
HNS → MC	5.42 (.66)	.67 (.48)	8.61 (84)			ns	3.80*** (1.51)	ns
HNS (no transition)	5.25 (1.02)	.37 (.15)	7.93 (1.89)			-2.19* (.50)	2.48* (1.75)	ns
HNS → HSA	4.98 (1.29)	1.32 (91)	7.80 (2.79)			**-**2.39* (.39)	7.13*** (1.76)	ns
**HSA** → **LSA**	**4.86 (2.55)**	**.80 (.88)**	**8.68 (1.96)**			-	-	-
HSA → MC	5.58 (.54)	.58 (.19)	8.73 (.48)			ns	ns	ns
HSA → HNS	4.83 (1.37)	.51 (.18)	7.41 (2.16)			ns	ns	-2.27* (.59)
HSA (no transition)	5.05 (1.83)	1.00 (.77)	7.37 (3.92)			ns	ns	-2.57* (.35)

Note. * p < .05, ** *p* < .01, *** *p* < .001. Transition to LSA is in bold as the reference group. LSA = Low social anxiety; MC = Moderate cognitive disturbance; HNS = High with difficulties in new situations; HSA = High social anxiety; PA: Peer adjustment; PV: Peer victimization; SWB = Subjective well-being.

Of the adolescents who were grouped in the LSA profile at T1, the ones who remained in this profile scored higher on subjective well-being than those who moved to the HNS profile at T2. For the MC profile at T1 no significantly different outcomes were found between the transitions. Of the adolescents who were grouped in the HNS profile at T1: (a) those who moved to the LSA profile had higher peer adjustment scores than those who moved to the HSA profile, and to those who moved to the HAS profile; (b) those who moved to the LSA profile had lower peer victimization scores than those who remained in the HNS profile, and to those who moved to the MC and the HSA profiles. Of the adolescents who were grouped in the HSA profile at T1: those who moved to the LSA profile had a higher subjective well-being than those who remained in the HSA profile, and to those who moved to the HNS profile. The effect size differences between the profile transitions were medium to high (see Table 3).

## Discussion

The present study aims to contribute to the literature by analyzing different longitudinal social anxiety profiles through Latent Transition Analysis (LTA) and by investigating the effects of profile transition on psychosocial adjustment. In line with Yu et al. (2020), it showed that adolescents could best be classified using heterogeneous and mixed patterns of social anxiety, rather than according to the traditional classification of cognitive, behavioral, and emotional responses. Using a large sample of Spanish adolescents, the present study is the first to analyze individual-level transition between social anxiety profiles over two time points and assess psychosocial implications of these dynamics.

Similar to Yu et al. (2020), in the present study a four-profile structure best fit at both time points. Adolescents were classified in profiles of: (1) low social anxiety, with low scores on all social anxiety items; (2) moderate cognitive disturbance, in which concerns about negative evaluation by others were prominent; (3) high with difficulties in new situations, with behavioral and emotional difficulties when facing new social situations; and (4) high social anxiety, characterized by relatively high levels of social anxiety on all dimensions. Finding a structure that largely matches that of Yu et al. (2020) supports that a person-centered approach that considers the heterogeneity of social anxiety is appropriate for understanding and studying the phenomenon, as it takes greater account of differences between adolescents. The robustness of this structure was confirmed through the measurement invariance analysis, which showed the same profile pattern was valid for both time points. Consistent with recent research (Gómez-Ortiz et al., 2020; Yu et al., 2020), girls, middle adolescents and those with higher demonstration-avoidance goals were more likely to fall within higher social anxiety profiles. These results are in line with three-dimensional theory (Lang, 1968), which emphasizes that social anxiety is characterized by response with different patterns.

The study also provides information about the stability of social anxiety patterns in adolescents over time. Most individuals were classified in the same profile at both time points, and adolescents who transitioned between profiles mostly moved to similar profiles. These findings are consistent with previous research in which the stable nature of social anxiety during adolescence was emphasized (Chiu et al., 2021). Only a small proportion, and more likely boys than girls, transitioned between the extreme profiles. Girls were more likely to remain within the same profile if they had been classified at T1 into the moderate cognitive disturbance and high social anxiety profiles. This suggests that patterns of social anxiety are more stable in girls. The age and the level of social demonstration-avoidance goals did not affect transitioning between social anxiety profiles.

The third aim was to contribute to the knowledge about potential maladaptive effects of social anxiety and to examine this with the same person-centered approach, distinguishing these effects for different profile transitions. Adolescents who moved to the MC profile exhibited an adverse result in terms of peer victimization if they came from the HNS profile in comparison with those adolescents who transitioned to the LSA profile. The lack of any other adverse outcomes shows that those adolescents who moved to moderate levels of fear of negative evaluation did not experience maladaptive psychosocial consequences in peer adjustment and subjective well-being. The high stakes social interaction model (Buttermore, 2009) argues that moderate levels of social anxiety can trigger an adaptive response to the social environment. Social anxiety may allow adolescents to trigger mechanisms to avoid exclusion, rather than to avoid feared situations, leading to a more speedy and accurate recognition of social threats. This social anxiety response can elicit physiological and psychological resources to respond in social interactions and to continue to be perceived as a valued member of the peer group. In this line, only high levels of social anxiety should be considered a disorder (Borg & Willoughby, 2021).

The results also showed that the low and moderate profiles in T1 were mostly not associated to peer and psychological problems in T2, and that it is those who remain or move within the higher social anxiety profiles who show worse outcomes in peer adjustment, subjective well-being, and peer victimization. Adolescents with such profile dynamics feel shy and unable to face new social situations, leading to a distressed adjustment to the context. Some adolescents may not achieve the same degree of social skills as their peers, which can result in a reduced development of positive interactions (Romera et al., 2021). An explanation for these relationships from a socio-cognitive perspective is that social anxiety could reduce learning opportunities and thus undermine the healthy development of peer interactions (Gazelle & Rubin, 2019), which can make them more vulnerable and likely targets of bullying (Chiu et al., 2021). Moreover, the peer adjustment results are in line with earlier findings that both socially anxious adolescents perceive themselves as being treated more negatively by classmates, and that classmates observe socially anxious adolescents to receive more negative treatment (Blöte et al., 2019). The maladaptive effects of social anxiety on peer adjustment and peer victimization are also reflected in the lower subjective well-being of adolescents in the transition to high anxiety profiles. It suggested that during adolescence most enjoyment is achieved when spending time with peers, but some may feel unable to express their needs, emotions or ideas for risk or fear of being rejected or shamed by their peers (Bravo et al., 2022).

This study has several limitations. First, it only includes self-report measures. Future studies could consider other sources of information, for example peer nominations or teachers' reporting for variables such as peer victimization and peer adjustment. Second, the effects of the profile transitions on maladaptive behavior and well-being were measured simultaneously with the second data point of the SAS-A. Future studies could explore whether these psychosocial implications persist when treated as a distal outcome at a later stage of the LTA or how the outcomes develop by taking a growth modelling approach. In the same line, it would be beneficial to test the dynamics over a longer period of time. Furthermore, the LTA was performed without controlling for the effects that developmental changes and transitions in adolescence, as stressors, may have on social anxiety. Examples of such stressors that would be beneficial to include in future studies could lie in the interpersonal domain, relating to the school environment (a change of school, a transition from middle to high school), peer relationships (the emergence of romantic relationships), and the family environment (parenting styles), but also include individual biological factors (physiological changes or changes in body morphology).

Despite these limitations, the study offers a significant contribution on several levels. As a theoretical contribution, the study explores heterogeneous social anxiety profiles in adolescents with a longitudinal design, with the aim to understand how the dynamics in social anxiety profiles relate to psychosocial adjustment in adolescence. The main conclusions from this study are: (a) social anxiety profiles are associated with cognitive, emotional, and behavioral responses and robust over time; (b) the stability of adolescents' profile classifications is moderate, being higher in girls, and with boys more likely to switch between extreme profiles in both directions; (c) many adolescents who remained in or transitioned to profiles with higher social anxiety showed poorer psychosocial adjustment. In short, looking at transition probabilities helps to better understand the development of social anxiety during adolescence and gives insights into its structure.

The study also contributes to the practice of dealing with adolescents' social anxiety, by underlining the importance of acknowledging adolescents’ characteristics in the development and choice of intervention. Adolescents in whom the social anxiety decreases and who remain with only moderate cognitive responses should be kept in focus as they may still need cognitive adjustment strategies (McLellan et al., 2015) to cope with possible victimization. For adolescents who persist in or move towards moderate fear of negative appraisal and high social avoidance and distress in new situations and in general, emphasis could first be put on improving their emotional and behavioral responses, since it has been suggested that moderate levels of cognitive disturbance can be adaptive. This could be done through the promotion of approach coping strategies and social skills in schools and families (Gómez-Ortiz, et al., 2019; Spence et al., 2017). Finally, adolescents whose social anxiety increase considerably or remain at a high level need to be treated using a preventive intervention that incorporates all three social anxiety dimensions to promote their psychosocial adjustment (La Greca & Lopez, 1998). Social anxiety interventions have focused mainly on treating clinical levels of social anxiety disorder using behavioral and cognitive-behavioral treatment models (Neufeld et al., 2020). Adding a more tailored approach by distinguishing different social anxiety profiles and its dynamics should improve the effectiveness of social anxiety interventions and reduce the negative effects on peer relationships and well-being.
